# A rapid method for quantification of persistent and mobile organic substances in water using supercritical fluid chromatography coupled to high-resolution mass spectrometry

**DOI:** 10.1007/s00216-020-02722-5

**Published:** 2020-06-10

**Authors:** Stefanie Schulze, Heidrun Paschke, Till Meier, Matthias Muschket, Thorsten Reemtsma, Urs Berger

**Affiliations:** 1 Department of Analytical Chemistry, Helmholtz Centre for Environmental Research - UFZ, Permoserstrasse 15, 04318 Leipzig, Germany; 2 Institute of Analytical Chemistry, University of Leipzig, Linnéstrasse 3, 04103 Leipzig, Germany

**Keywords:** Persistent and mobile organic substances, PM substances, PMOCs, Supercritical fluid chromatography, Evaporation, Water samples

## Abstract

Persistent and mobile organic substances (PM substances) are a threat to the quality of our water resources. While screening studies revealed widespread occurrence of many PM substances, rapid trace analytical methods for their quantification in large sample sets are missing. We developed a quick and generic analytical method for highly mobile analytes in surface water, groundwater, and drinking water samples based on enrichment through azeotrope evaporation (4 mL water and 21 mL acetonitrile), supercritical fluid chromatography (SFC) coupled to high-resolution mass spectrometry (HRMS), and quantification using a compound-specific correction factor for apparent recovery. The method was validated using 17 PM substances. Sample preparation recoveries were between 60 and 110% for the vast majority of PM substances. Strong matrix effects (most commonly suppressive) were observed, necessitating a correction for apparent recoveries in quantification. Apparent recoveries were neither concentration dependent nor dependent on the water matrix (surface or drinking water). Method detection and quantification limits were in the single- to double-digit ng L^−1^ ranges, precision expressed as relative standard deviation of quadruplicate quantifications was on average < 10%, and trueness experiments showed quantitative results within ± 30% of the theoretical value in 77% of quantifications. Application of the method to surface water, groundwater, raw water, and finished drinking water revealed the presence of acesulfame and trifluoromethanesulfonic acid up to 70 and 19 μg L^−1^, respectively. Melamine, diphenylguanidine, *p*-dimethylbenzenesulfonic acid, and 4-hydroxy-1-(2-hydroxyethyl)-2,2,6,6-tetramethylpiperidine were found in high ng L^−1^ concentrations.

**Electronic supplementary material:**

The online version of this article (10.1007/s00216-020-02722-5) contains supplementary material, which is available to authorized users.

**Figure:**
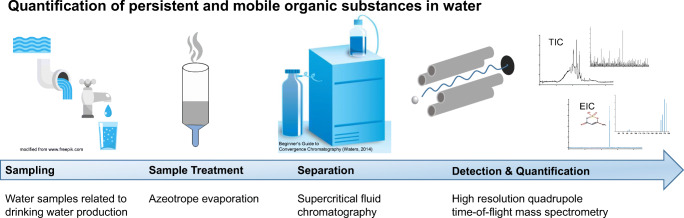
Graphical abstract

## Introduction

Persistent and mobile organic substances (PM substances, also referred to as PMOCs) are characterized by a high environmental stability and a very low potential to sorb to surfaces [1, 2]. PM substances that are emitted into the environment [3] would thus partition to and stay in the water phase and penetrate natural (bank filtration, subsurface passage) and technical (wastewater treatment plants, drinking water treatment) barriers in water cycles. Therefore, PM substances are of concern regarding the quality of our drinking water resources [4].

The characteristic of high aquatic mobility makes PM substances hard to analyze using common reversed phase liquid chromatography (RPLC) techniques [1, 5]. Since hydrophobic interactions are the driving force of retention in RPLC, highly polar and thus in-water-mobile compounds are not retained and elute in the void volume together with very polar matrix constituents. In recent years, alternative separation techniques were developed for retention and separation of highly mobile substances [5], including ion chromatography [6], hydrophilic interaction liquid chromatography (HILIC) [7, 8], mixed-mode liquid chromatography (MMLC) [9, 10], and supercritical fluid chromatography (SFC) [11, 12].

SFC as a separation technique was first reported by Klesper and co-workers [13] in 1962. Since then, the number of reports on applications of SFC is continuously increasing [14, 15]. SFC is often described as an alternative to normal-phase chromatography and as the method of choice for enantioselective separations, particularly for non-volatile compounds [16]. However, due to the possibilities of using reversed phase as well as normal-phase stationary phases and to mix a polar co-solvent into supercritical (non-polar) CO_2_ in the mobile phase, SFC is a very versatile separation technique, even encompassing applications for highly polar and mobile analytes [17]. Recent examples include environmental water pollutants [18, 19], polar urinary metabolites [20], and polar compounds in anti-doping control [21]. Desfontaine and co-workers [22] compared matrix effects in SFC and RPLC coupled to tandem mass spectrometry for analysis of doping agents and pharmaceuticals in urine. They found that SFC generally led to lower matrix effects than RPLC, especially when applying a simple dilute-and-shoot protocol [22].

Besides chromatography, the extraction and enrichment of PM substances from water samples also poses a challenge [5]. Enrichment is necessary, since direct injection of water samples into the analytical instrument [23] is often not sensitive enough for detection of trace levels in samples from background areas. Additionally, large-volume injection of water (> 10 μL) is not compatible with SFC. Solid-phase extraction is the most commonly used method for enrichment of contaminants from water samples. However, retention of PM substances on common SPE material is usually poor (again due to the high mobility) or very specific, such as, e.g., for negatively charged PM substances on an anion exchange resin [10]. Evaporation [7] or freeze-drying [9] are more generic methods for analyte enrichment with the disadvantage that all non-volatile constituents in the sample are quantitatively enriched as well, which may lead to significant matrix effects.

In a recent study, we have applied innovative analytical methods for a qualitative screening study of PM substances in environmental water samples [11]. Out of 57 target analytes, 43 (75%) were detected in surface water and/or groundwater samples. This high detection percentage underlines the importance of being able to quantify PM substances in different types of environmental waters, including drinking water. For ion chromatography, HILIC, and MMLC, quantitative methods have been explored for PM substances (see above), but not for SFC so far. The aim of the present study was thus to develop and validate a trace analytical method based on SFC coupled to high-resolution mass spectrometry as an alternative and potentially complimentary method for quantitative analysis of a variety of PM substances in environmental as well as in drinking water samples.

## Experimental section

### Chemicals and reagents

**Fig. 1:**
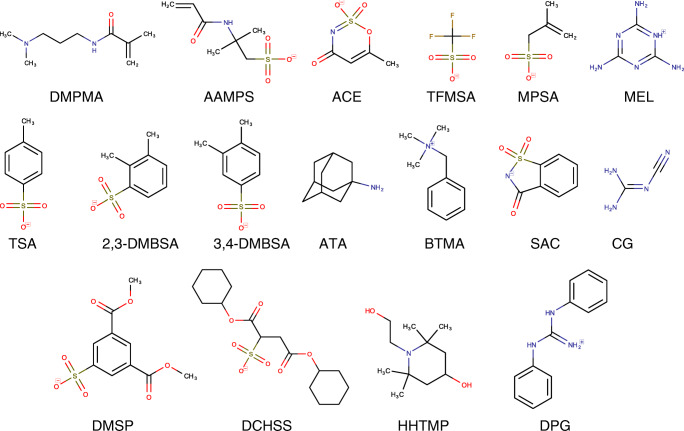
The 17 selected PM substances with their acronyms used in the present study. For full names and CAS numbers, see ESM Table S1. Ionizable substances are shown in their charge state at a pH value of 7

For method development and validation, 17 model PM substances were selected (Fig. 1) from a list of PM substances detected in environmental water samples in a qualitative screening study [11]. The target PM substances were selected to span a broad range of (yet very low) log *D* values (estimated at pH 7 to − 3.06 to 1.23, ChemAxon, JChem for Office, version 19.26.0.571), molecular masses (84 to 361 g mol^−1^), and charge states, as detailed in Table S1 in the Electronic Supplementary Material (ESM). Stock standard solutions of the analytes were prepared at 1 mg mL^−1^ in acetonitrile or water (depending on solubility) and stored in darkness at − 20 °C. From these stock solutions, mixture solutions of the 17 PM substances at different concentration levels were prepared. All used chemicals, solvents, and reagents were of analytical grade (ESM Table S2).

### Water samples

For method development and validation, surface water samples from the rivers Götsche and Mulde (near Halle and Leipzig, Germany) and drinking water samples from the tap in our laboratory were used. For method application, six water samples were obtained from two different regions in Germany (South Hessia and Berlin) including surface water, groundwater, and water from drinking water treatment plants (for details on the samples, see ESM Table S3). The samples from the drinking water treatment plant Tegel were taken and analyzed with permissions from the drinking water company (Berliner Wasserbetriebe). Sampling took place between 2017 and 2019. The samples were stored up to 2 weeks at + 4 °C until analysis.

### Sample preparation

The samples were filtered through a glass fiber filter (see ESM Table S2 for details on materials and instrumentation used in sample preparation). Azeotrope evaporation (AZEVAP) was used as enrichment procedure. An aliquot of 4 mL of the filtered sample was mixed with 21 mL acetonitrile (ratio for the minimum azeotrope mixture) in an evaporation glass vial with a tip in the bottom. This mixture was evaporated to dryness at 40 °C under a stream of argon, while the glass walls were repeatedly rinsed with acetonitrile to make sure that the residue concentrated in the tip. The residue was reconstituted in 100 μL acetonitrile:Milli-Q water (90:10), resulting in a sample-to-extract enrichment factor of 40. In case of precipitation, the extract was filtered through a lint-free paper wipe covering the tip of a Pasteur pipette while it was transferred into an autoinjector vial.

### Instrumental analysis

SFC (Waters Acquity UPC^2^ system) was performed on a BEH column (for analyses in positive ion mode) or Torus Diol column (for analyses in negative ion mode) coupled to quadrupole time-of-flight high-resolution mass spectrometry (HRMS; Waters Synapt GS2) (ESM Table S2). Aliquots of 10 μL of the sample extracts were injected. Separation was performed at 55 °C at a flow rate of 1.5 mL min^−1^ using a carbon dioxide-methanol/water gradient containing 0.2% ammonium hydroxide in the methanol/water co-solvent (ESM Fig. S1A). A methanol/water make-up flow containing 0.1% formic acid was used at 0.3 mL min^−1^ to transfer the column effluent into the mass spectrometer. The HRMS instrument was operated in positive or negative electrospray ionization (ESI) and full scan mode (*m*/*z* 50 to 600) at a resolution of 20,000. Mass calibration on a mass range of *m*/*z* 50 to 1200 was performed using a calibration solution to generate 17 reference masses in positive and 16 in negative ionization mode. A root mean square residual mass error < 1 ppm was obtained. During measurements in both ionization modes, a lock-spray containing leucine enkephalin was continuously infused. Two ions were selected for identification of the PM substances (except for MPSA and CG that produced only one ion), and the most intense ion was used for quantification (ESM Table S4). A mass tolerance of 5 ppm was used when extracting high-resolution mass chromatograms of the analytes.

As a reference for SFC separation and retention, commonly used RPLC based on a C_18_ stationary phase (Waters Acquity UPLC HSS T3 column) was used (ESM Table S2). The RPLC was coupled to triple quadrupole tandem mass spectrometry (MS/MS). Aliquots of 10 μL of the sample extracts were injected in water. Separation was performed at 60 °C at a flow rate of 500 μL min^−1^ using a water/methanol gradient containing 5 mM ammonium formate (ESM Fig. S1B). The mass spectrometer was operated in positive/negative-switching ESI mode. Scheduled multiple reaction monitoring (MRM) mode was applied, acquiring two transitions for each analyte (ESM Table S5).

### Quantification

Quantification was performed using an external 5-point calibration curve in pure solvent and applying a compound-specific correction factor for the apparent recovery (corresponding to a matrix- and method-matched calibration). The calculation of the apparent recovery is explained in the section “Apparent recoveries (sample preparation recoveries and matrix effects)”, and the applied values are listed in ESM Table S6. The correction factor was calculated from the mean of the apparent recoveries determined in different experiments (varying in spike concentrations and water matrices), as neither the spike concentration nor the water matrix (surface or drinking water) had a significant influence on the apparent recovery (see “Results and discussion” section below).

### Method performance validation

We validated the method by determining instrumental blanks, instrumental detection limits, linear range of detection, apparent recoveries (i.e., sample preparation recoveries and matrix effects), procedural blanks, method detection and quantification limits, and accuracy (i.e., precision and trueness).

#### Instrumental blanks, instrumental detection limits, and linearity

Instrumental blank contamination was evaluated by solvent injections (acetonitrile:Milli-Q water 90:10) into the SFC-HRMS system. Instrumental detection limits (IDLs) and the linear range of detection were determined using a dilution series (*n* = 10) of the standard mixture (consisting of the 17 PM substances) covering a concentration range of 0.05–500 ng mL^−1^. The coefficient of determination (*R*^2^) for linear regression was calculated. IDLs were set for each PM substance to the injected amount, leading to a signal in the extracted high-resolution mass chromatogram with a signal-to-noise ratio of at least 3. In case of instrumental blank contamination, the IDL was calculated from the quantified signal areas in 10 solvent blank injections based on mean plus 3 times standard deviation of the signal areas in the 10 blanks.

#### Apparent recoveries (sample preparation recoveries and matrix effects)

Sample preparation recovery and matrix effect experiments were performed using surface water from the river Götsche and drinking water from the tap in the laboratory (ESM Table S3). All experiments were performed in triplicates and analyzed by SFC-HRMS. Each PM substance was spiked at two to three different concentrations in both water matrices (ESM Table S6) before and after enrichment. Spike concentrations differed between the PM substances based on the differences in IDLs. Additionally, both water matrices were also enriched and analyzed without spiking. Areas of PM substances in the chromatograms of the non-spiked samples were subtracted from areas in the chromatograms of the respective spiking experiments (→ netArea).

The sample preparation recovery (Recov) was calculated according to Eq. (1)

1 $$\mathrm{Recov}\ \left(\%\right)=\left(\frac{{\mathrm{netArea}}_{\mathrm{PM}\ \mathrm{substance}\ \mathrm{spiked}\ \mathrm{before}\ \mathrm{enrichment}}}{{\mathrm{netArea}}_{\mathrm{PM}\ \mathrm{substance}\ \mathrm{spiked}\ \mathrm{after}\ \mathrm{enrichment}}}\right)\times 100$$

The matrix effect (ME) in ionization was calculated according to Eq. (2)

2 $$\mathrm{ME}\ \left(\%\right)=\left(\frac{{\mathrm{netArea}}_{\mathrm{PM}\ \mathrm{substance}\ \mathrm{spiked}\ \mathrm{after}\ \mathrm{enrichment}}}{{\mathrm{Area}}_{\mathrm{PM}\ \mathrm{substance}\ \mathrm{in}\ \mathrm{pure}\ \mathrm{solvent}}}\right)\times 100-100$$

Finally, the apparent recovery (combination of Recov and ME) was calculated according to Eq. (3)

3 $$\mathrm{Apparent}\ \mathrm{recovery}\ \left(\%\right)=\left(\frac{{\mathrm{netArea}}_{\mathrm{PM}\ \mathrm{substance}\ \mathrm{spiked}\ \mathrm{before}\ \mathrm{enrichment}}}{{\mathrm{Area}}_{\mathrm{PM}\ \mathrm{substance}\ \mathrm{in}\ \mathrm{pure}\ \mathrm{solvent}}}\right)\times 100$$

Further, matrix effects on the chromatography were assessed qualitatively by comparison of chromatograms (retention times and signal shape) from standards in pure solvent and from spiked extracts of environmental water samples.

#### Procedural blanks and method detection and quantification limits

Procedural blank experiments were performed by applying the full sample preparation procedure but without any water matrix in the enrichment step (i.e., starting from 21 mL pure acetonitrile). Five replicates of procedural blanks were prepared. The method detection limit (MDL) and method quantification limit (MQL) were determined by spiking surface water and drinking water samples at two to three different concentrations per analyte (ESM Table S6) and quantifying them according to the described protocol. The signal-to-noise ratios were recorded, and the quantified concentrations were extrapolated (from a signal with a signal-to-noise ratio close to 10) to a signal-to-noise ratio of 3 (MDL) or 10 (MQL). In case of procedural blank contamination, the MDL and MQL were calculated from the quantified signal areas in the procedural blank chromatograms based on mean signal area plus 3 times (MDL) or 10 times (MQL) standard deviation.

#### Accuracy (precision and trueness)

For evaluation of precision and trueness, the following set of experiments (independent from the earlier experiments for determination of sample preparation recoveries and matrix effects) was performed. All PM substances were spiked (*n* = 4) into surface water from the river Götsche (see ESM Table S7 for compound-specific spiking levels). From these experiments (including non-spiked river Götsche water), the apparent recoveries and correction factors were calculated as described before. The PM substances were also spiked into surface water from the river Mulde and into drinking water (each *n* = 4, the spiking concentrations for Mulde water were 0.3 times and those for drinking water were 0.03 times the concentrations spiked to water from river Götsche; see ESM Table S7). All samples were analyzed and quantified using the correction factors determined for river Götsche. The quantified concentrations were corrected with levels determined in the corresponding non-spiked samples. The relative standard deviations of the quantification (*n* = 4) were used as a measure of method precision. To assess trueness, the averaged quantified concentrations were compared to the theoretical (spiked) concentrations.

## Results and discussion

### Enrichment method for PM substances

The most commonly used enrichment method for organic trace pollutants from water samples is SPE. SPE has also been used in two methods for PM substances published earlier [7, 10]. However, SPE sorbents are usually designed to selectively retain certain groups of chemicals (e.g., only anions or only cations). Therefore, Zahn and co-workers [7, 24] developed their own homemade SPE cartridges from three different sorbents while Montes et al. [10] subjected each water sample to two different SPE procedures. We attempted to develop a quick and generic method for a broad range of PM substances, which can be used in larger screening or monitoring programs. Therefore, we used AZEVAP, which requires very little sample handling and is applicable to all analytes, but also leads to enrichment of all other non-volatile constituents in the samples. Any generically applicable enrichment method would inherently also enrich the majority of matrix compounds.

### SFC-HRMS method development

#### Selection of stationary and mobile phase

In the development of the SFC method, four different stationary phases and four modifiers in the co-solvent of the mobile phase were tested in a 4 × 4 matrix. The tested columns (stationary phases) included Torus Diol, Torus 2-PIC, BEH, and BEH 2-EP (all from Waters), which can be classified as normal phase or hybrid stationary phases. The mobile phase consisted of supercritical CO_2_ and a methanol/water co-solvent (ESM Fig. S1A) with formic acid, ammonium hydroxide, ammonium formate, or ammonium acetate as modifier. Ammonium hydroxide and formic acid slightly improved peak shape and response for most PM substances and were superior to the other two tested modifiers. Ammonium hydroxide was chosen for the final method and added at an optimized ratio of 0.1% to the co-solvent. In terms of stationary phases, BEH (a hybrid stationary phase showing both reversed and normal phase characteristics) and Torus Diol (a normal phase) showed the best performances. Regarding chromatography, all analytes were well retained (see ESM Table S4 for retention factors) and showed sharp peaks (see the section “Retention time stability and influence of sample matrix on the chromatography”) on both of these columns. However, while PM substances that were recorded in positive ionization mode generally showed a slightly better response after separation on the BEH column, Torus Diol led to slightly more sensitive detection for analytes in negative ionization mode. Both columns were thus used in the final method, one for each polarity of mass spectrometric detection. For a higher sample throughput with polarity-switching MS, any of the two columns could be used without a substantial loss of sensitivity.

#### Effect of sample diluent and injection volume

**Fig. 2:**
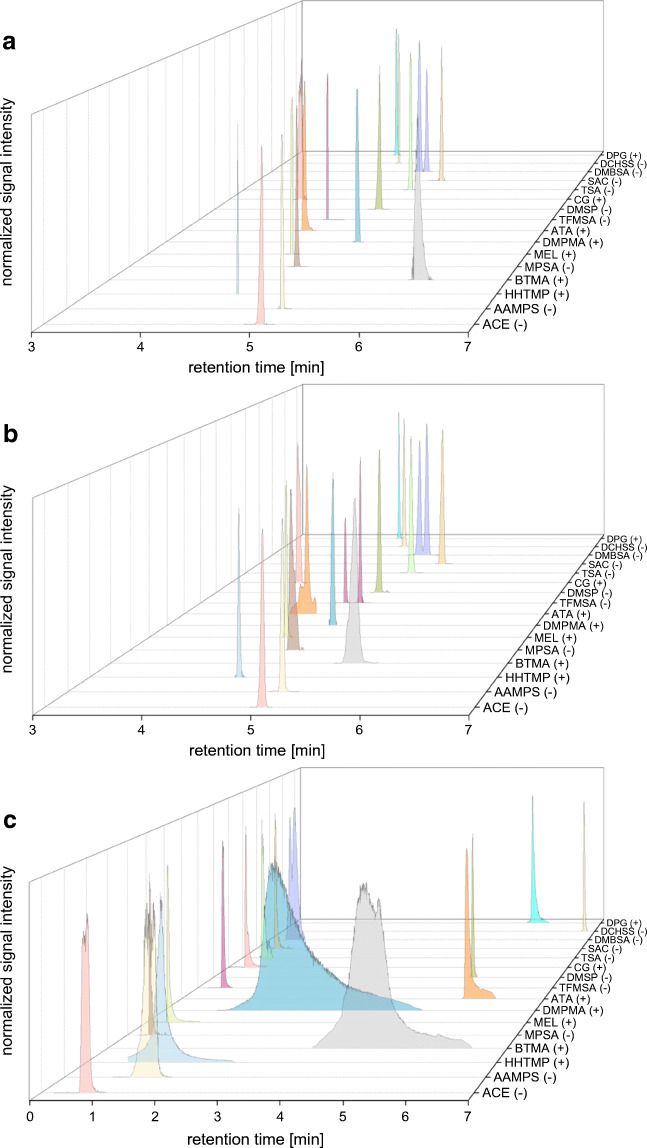
SFC-HRMS extracted mass chromatograms of a standard mixture of the target PM substances sorted by log *D***a** injected in acetonitrile:water 90:10 except for TFMSA (in pure water) and **b** spiked to surface water and, after extraction, injected in acetonitrile:water 90:10 for all compounds. (+) indicates BEH chromatography and detection in positive ion mode, and (−) indicates Torus Diol chromatography and detection in negative ion mode. The two peaks for DMBSA are the chromatographically resolved 3,4-isomer and 2,3-isomer. For comparison, panel **c** shows the RPLC-MS/MS-extracted MRM chromatograms of a standard mixture injected in Milli-Q water. Note the different retention time scales between the SFC and the RPLC chromatograms

The composition of the injection solvent as well as the injection volume play an important role in SFC, affecting both peak shape and intensity [25, 26]. A good compromise between compound solubility, SFC compatibility, sensitivity, and peak shape needs to be found in a multi-analyte method. In the present study, different solvent compositions of acetonitrile and Milli-Q water (from 0 to 100% acetonitrile) were tested. Peak shapes and signal intensities varied considerably depending on diluent composition. Pure Milli-Q water provided broad peaks and low intensity for all of the tested chemicals, with the exception of TFMSA. TFMSA showed a very sharp peak when injected in water (Fig. 2a), but a split peak in the presence of acetonitrile (Fig. 2b). An explanation for this peculiar behavior of TFMSA was not found. The best compromise considering all tested PM substances was acetonitrile:water 90:10 as injection solvent, providing better peak areas than pure acetonitrile and good peak shapes (Fig. 2a, with the exception of TFMSA). The high proportion of 90% acetonitrile also allowed using the maximum possible injection volume of 10 μL provided by the instrument without compromising peak sharpness. With these settings, TFMSA showed a split peak for both standards and samples (Fig. 2b) and was integrated as the sum of the two signal areas.

#### Retention time stability and influence of sample matrix on the chromatography

As can be seen from Fig. 2a and b, matrix effects on chromatographic retention resulting from the surface water matrix were only observed for BTMA with a shift to a shorter retention time. The relative standard deviation (*n* = 10) of the retention times for all PM substances analyzed over a couple of days was < 0.1% in both standard mixtures and sample extracts. An important requirement for reproducible retention times was, however, a freshly prepared co-solvent with modifier (at least every other day). The co-extracted matrix had an effect of slight peak broadening on some target analytes, which was most pronounced for MPSA and ATA (Fig. 2a, b).

#### Comparison with RPLC

For comparison of chromatographic method performance, a RPLC-MS/MS method using the MRM mode was also developed for the 17 target PM substances (see ESM, Tables S2 and S5 and Fig. S1). Chromatography was optimized to obtain the best possible retention on the C_18_ column for a maximum of analytes. For this purpose, three different columns (two polar-modified C_18_ materials and a porous graphitic carbon column) were tested with formic acid, ammonium formate, or diethyl amine in the mobile phase. Anyhow, in the optimized system (described in the “Experimental section”), 8 of the PM substances eluted very close to or in the void volume, preventing complete separation of the DMBSA isomers (Fig. 2c). Of the 9 retained substances, 6 showed very poor peak shapes. Only DMSP, DCHSS, and DPG showed good retention and sharp peaks in RPLC, the latter two being the PM substances with the highest log *D* values among the tested analytes. In comparison, the SFC method is clearly superior to the RPLC method in terms of peak shapes and retention, which considerably facilitates signal detection and integration (compare Fig. 2a and c). Furthermore, SFC was able to separate the two isomeric compounds (3,4-DMBSA and 2,3-DMBSA) (Fig. 2a, b). While RPLC showed a slight tendency towards higher retention for analytes with higher log *D* values (Fig. 2c, ESM Table S5), no association between log *D* value and retention factor could be observed in SFC.

### Method performance validation

#### Instrumental blanks, instrumental detection limits, and linearity

HHTMP, MEL, TFMSA, and DPG were repeatedly detected in instrumental blanks. In the present study, no effort was made to elucidate or eliminate the sources of these background contaminations. However, instrumental blanks were considered in the determination of IDLs, as detailed in the “Experimental section.” SFC-HRMS-based IDLs are summarized in Table 1 for all studied PM substances and range between 0.1 and 5 pg for SFC with BEH and between 0.02 and 10 pg for SFC with Torus Diol. Good linearity of the instrumental method was observed for all analytes over at least 3 orders of magnitude (with the exception of the guanidines CG and DPG with smaller linear ranges) with correlation coefficients (*R*^2^) higher than 0.99 (Table 1) and residuals < 25%.

**Table 1:** Instrumental detection limits (IDLs), linear ranges with correlation coefficients (*R*^2^), as well as method detection and quantification limits (MDLs/MQLs) for the analysis of 17 PM substances by SFC-HRMS

Analyte	IDL_BEH_ (pg)	Linear range, ng mL^−1^ (*R*^2^)	IDL_Torus Diol_ (pg)	Linear range, ng mL^−1^ (*R*^2^)	MDL/MQL	
					AZEVAP–BEH (ng L^−1^)	AZEVAP–Torus Diol (ng L^−1^)
ACE (−)	0.6	–	0.5	0.05–500 (0.998)	–	14/33
AAMPS (−)	0.2	–	0.3	0.05–75 (0.998)	–	10/30
HHTMP (+)	0.2	0.05–15 (0.998)	0.09	–	5/14	–
BTMA (+)	0.3	0.05–150 (0.999)	0.3	–	3/10	–
MPSA (−)	3	–	0.4	0.5–150 (0.997)	–	50/90
MEL (+)	0.3	0.05–75 (0.998)	4	–	4/10	–
DMPMA (+)	0.3	0.05–75 (0.999)	0.6	–	3/10	–
ATA (+)	0.2	0.5–150 (0.995)	4	–	2/5	–
TFMSA (−)	0.1	–	0.05	0.05–150 (0.998)	–	4/5
DMSP (−)	0.1	–	0.1	0.05–150 (0.999)	–	10/30
CG (+)	5	5–75 (0.990)	10	–	30/61	–
TSA (−)	0.5	–	0.2	0.05–300 (0.997)	–	14/31
SAC (−)	0.8	–	0.8	0.5–500 (0.993)	–	15/30
3,4-DMBSA (−)	1	–	0.5	0.05–150 (0.998)	–	5/8
2,3-DMBSA (−)	0.1	–	0.02	0.05–500 (0.998)	–	26/42
DCHSS (−)	0.3	–	0.08	0.05–75 (0.996)	–	10/30
DPG (+)	0.3	0.05–15 (0.999)	0.3	–	33/71	–

#### Apparent recoveries

**Fig. 3:**
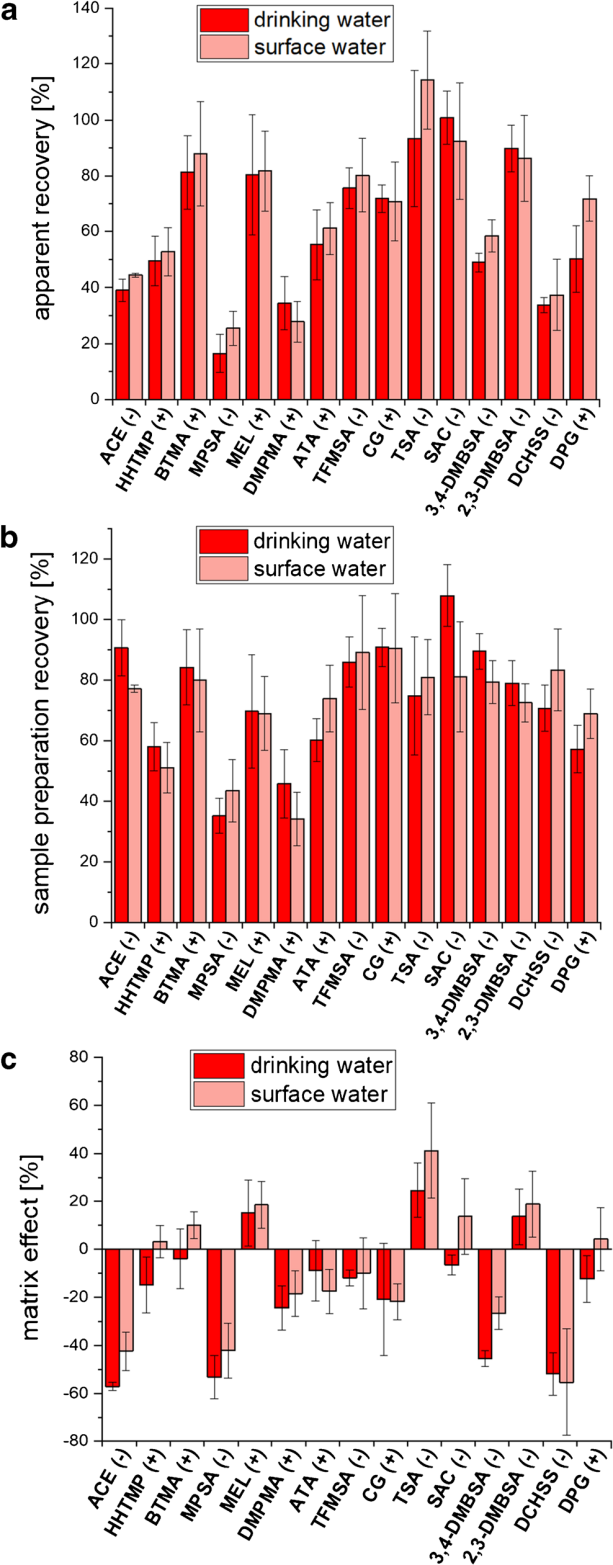
**a** Apparent recoveries, **b** sample preparation recoveries, and **c** matrix effects of the target PM substances spiked at different concentrations (see ESM Table S6) into drinking water (DW) and surface water (SW). Error bars indicate standard deviations (*n* ≥ 3). (+) or (−) indicates if the analyte was detected in positive or negative ion mode, respectively

For the vast majority of investigated PM substances, isotope-labeled analogues that could be employed as internal standards are not commercially available. Therefore, an external quantification method needed to be developed. Apparent recoveries, i.e., the combination of sample preparation recoveries and matrix effects, were investigated in order to evaluate if an external calibration curve of standards in pure solvent could be applied. For AAMPS and DMSP, the spiking concentrations were too low to reliably determine apparent recoveries. These two PM substances were excluded from further quantitative work, but still these were analyzed qualitatively. For all other analytes, apparent recoveries varied considerably, as representatively illustrated for one drinking water sample and one surface water sample in Fig. 3a. Thus, the quantification procedure had to include a correction for apparent recoveries. However, the apparent recoveries did not differ significantly between the drinking water and the surface water (Fig. 3a), nor were they concentration dependent. This observation was later confirmed during the accuracy testing using a different set of surface water and drinking water samples (see below). Therefore, an average compound-specific correction factor was calculated from both sample types at all tested concentrations and used in the quantification (ESM Table S6). The apparent recoveries for ACE and SAC obtained in our study were comparable with previously reported recoveries from water samples, e.g., by Tran et al. [27] or Montes et al. [10]. Tran and co-workers [27] used SPE enrichment with subsequent LC-MS/MS analysis. Montes at al. [9, 10] further published an apparent recovery for DPG (80%) from water samples after enrichment using a cation exchanger and analysis by MMLC-HRMS or LC-MS/MS, which is higher than our apparent recovery for DPG (61%) (Fig. 3a, ESM Table S6).

#### Sample preparation recoveries

Sample preparation recoveries and matrix effects were investigated independently to better understand the variability in apparent recoveries. Sample preparation recoveries were between 60 and 110% for the vast majority of PM substances for both water types (Fig. 3b) and could thus not (fully) explain the partially very low apparent recoveries (see, e.g., ACE or DCHSS in Fig. 3a and b).

#### Matrix effects

Matrix effects were an important reason for non-quantitative apparent recoveries in the present study, presumably mainly influencing the ESI process. Matrix effects are depicted in Fig. 3c as relative deviation of the signal area in the chromatogram of a spiked sample extract compared to a standard in pure solvent. Despite good retention of all analytes in SFC (Fig. 2a, b), strong suppression of the chromatographic signal by matrix was observed for 4 analytes (ACE, MPSA, 3,4-DMBSA, DCHSS) in both water matrices (Fig. 3c). Consistent signal enhancement with up to + 41% was only observed for 3 PM substances (MEL, TSA, 2,3-DMBSA). Matrix effects were largely comparable for the two different types of water. In an earlier study by Montes et al. [10] based on weak anion exchange or weak cation exchange enrichment of PM substances from water samples and MMLC-MS/MS analysis, very strong matrix suppression was also observed. However, Montes and co-workers [9, 10] also frequently observed matrix enhancement with up to + 150%. Thus, SPE does not necessarily produce “cleaner” extracts that lead to less matrix effects than the generic AZEVAP method. This finding is corroborated by the results by Köke and co-workers [24], who compared matrix effects between PM substances spiked to water extracts enriched by mixed-mode SPE and the same substances spiked to water extracts enriched by evaporation, in both cases analyzed by HILIC-MS/MS. The results showed that for 9 of 26 investigated substances (35%), matrix effects exceeded + 50% or − 50% in the SPE extracts, while this fraction was only 2/26 (8%) in evaporation extracts. Svan et al. [28] compared matrix effects on a range of pharmaceuticals between SFC-HRMS and RPLC-HRMS for a variety of matrices, including wastewater influent and effluent. They concluded that in both techniques, strong matrix effects occurred. In RPLC-HRMS, signal enhancements were commonly observed, while SFC-HRMS more often led to signal suppression. Signal suppression for the vast majority of analytes was also observed in our study (Fig. 3c).

Mitigation of matrix effects (i.e., separation of analytes from matrix constituents) in analysis of PM substances in water samples is inherently extremely challenging, since PM substances possess very similar physical-chemical properties as other organic constituents in water (i.e., dissolved organic matter). In our quantification method, we correct for matrix effects (as well as for sample preparation recoveries) by applying a compound-specific correction factor based on the observed apparent recoveries (ESM Table S6). This approach corresponds to using a matrix- and method-matched calibration and can easily be applied to large sample sets. Another (potentially more accurate but also much more laborious) approach would be the standard addition method, which results in compound-, method-, and even sample-specific corrections of apparent recoveries. Alternatively, standard addition over final extracts can be performed to correct for matrix effects, but not for sample preparation recoveries [10]. To simplify quantification methods (and to improve their precision, trueness, and comparability), synthesis of stable isotope-labeled internal standards for the most important PM substances should be envisaged.

#### Procedural blanks and method detection and quantification limits

Seven of the target compounds were detected in procedural blank samples. These were the PM substances already present in instrumental blanks (HHTMP, MEL, TFMSA, and DPG) as well as TSA, 3,4-DMBSA, and 2,3-DMBSA. Such procedural blank contamination was also observed in our earlier qualitative screening study [11]. These substances are high-production volume industrial chemicals (all > 100 t year^−1^) mainly used as plasticizers, as processing aids in polymers, and as vulcanization agents in polymerization processes. It is not unlikely that trace level contamination with such chemicals occurs from labware, like SPE cartridges, pipette tips, sealing, and tubing, or from solvents and reagents applied in the analytical method, though we did not attempt to elucidate the specific sources of contamination for the different analytes.

Comparable MDLs and MQLs were found for both water types (DW and SW), and the values are thus presented in Table 1 independent of the water matrix. MDLs and MQLs are typically in the low ng L^−1^ range, with MDLs ranging from 2 to 50 ng L^−1^ and MQLs from 5 to 90 ng L^−1^, depending on the compound. Our MDLs and MQLs are generally in the same range as values reported for PM substances analyzed by SPE enrichment and MMLC-MS/MS [10]. For the sweeteners acesulfame and saccharin, previous studies reported MQLs of 0.1 ng L^−1^ [29] or 25 ng L^−1^ [30], which are lower or similar compared to our study (see Table 1). However, it must be taken into account that the literature data was based on tandem MS quantification in selected reaction monitoring mode, which is more sensitive than detection by full-scan HRMS.

#### Accuracy (precision and trueness)

**Fig. 4:**
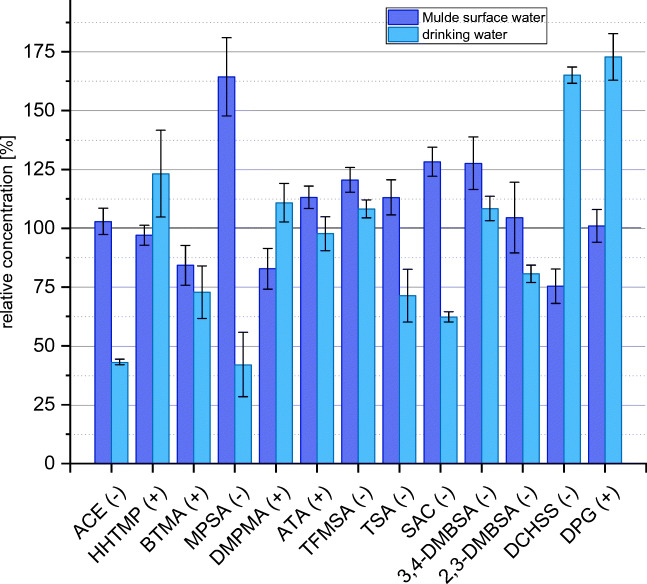
Accuracy experiments. Precision (error bars indicate the standard deviation of quantification, *n* = 4) and trueness (mean value of *n* = 4 relatively to theoretical (spiked) concentrations indicated as 100%) for analyte quantification in the spiked river Mulde and drinking water samples (after subtraction of levels present in the non-spiked samples). For calculation of the correction factors, the spiked river Götsche water samples were used. Spiking levels for river Mulde and drinking water were 0.3 and 0.03 times the levels of river Götsche. See ESM Table S7 for actual spiking concentrations and for numerical results

The results of the precision and trueness evaluation are given in Fig. 4 and ESM Table S7. The spiking concentration for river Mulde and drinking water samples was chosen to reflect actual levels occurring in surface or tap water, while for river Götsche, higher concentrations were chosen to reliably determine the correction factors for apparent recoveries (ESM Table S7). The correction factors determined earlier (ESM Table S6) were not used in this experiment, as there were several months between the first apparent recovery experiments (ESM Table S6) and the accuracy experiments (ESM Table S7) and compound-specific correction factors can change over time, potentially due to fluctuations in instrumental (ionization) performance. We therefore recommend to calculate apparent recoveries and correction factors for every sampling campaign. For MEL and CG, accuracy experiments could not be performed, due to the high concentration of MEL already present in the non-spiked river Götsche and due to the very small linear range of detection for CG (see Table 1). For all other target PM substances, precision, expressed as relative standard deviation of 4 replicate quantifications of spiked water samples (ESM Table S7), was in the range 4–14% (average ± standard deviation 8.5 ± 3.6%) for river Mulde and 2–32% (average ± standard deviation 9.8 ± 8.2%) for drinking water. These values are in the same range as the precision reported by Montes et al. [10] for a method based on mixed-mode SPE enrichment and mixed-mode liquid chromatography coupled to tandem MS. Trueness, expressed as percentage deviation of the quantified concentration (applying the correction factor from ESM Table S7) from the theoretical (spiked) concentration, was in the range − 25 to + 64% (average ± standard deviation 8.8 ± 22.6%) for river Mulde and − 58 to + 73% (average ± standard deviation − 3.2 ± 39.4%) for drinking water. The lower accuracy (lower precision and lower trueness) for the drinking water compared to river Mulde samples is most likely due to the 10 times lower concentrations spiked to drinking water compared to river Mulde (see the section “Accuracy (precision and trueness)” in the “Experimental section”). The excellent average values for trueness of + 8.8 and − 3.2% show that there is no systematic bias in the quantification method. Trueness reported for the method by Montes et al. [10] for individual PM substances was slightly better than that in our study. However, Montes and co-workers [9, 10] used a different set of test compounds and a sample-specific “standard addition over the extracts methodology” for quantification, which requires an individual calibration curve for each sample. Our method does not rely on sample-specific standard addition and is thus applicable to larger screening or monitoring studies.

### Method application to environmental water samples

**Fig. 5:**
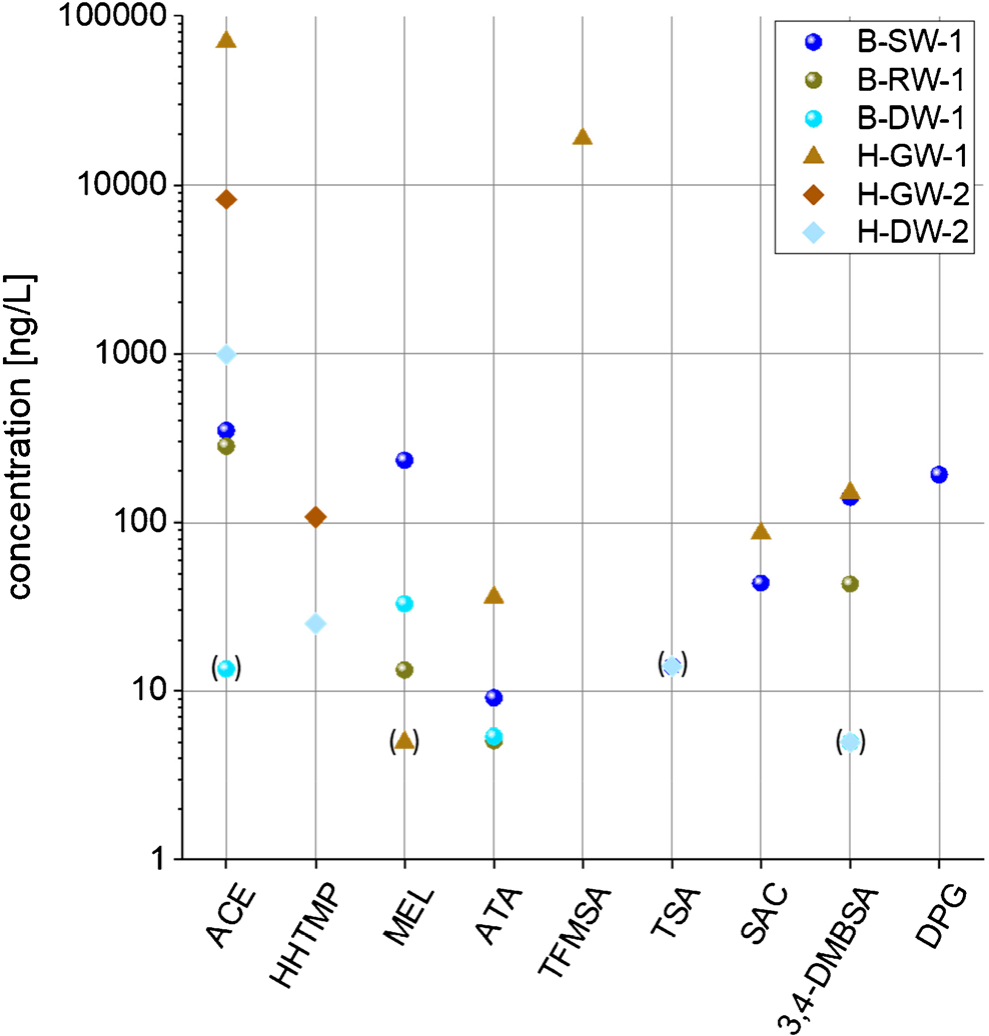
Concentration data of PM substances in environmental and drinking water samples. Estimated concentrations between the MDL and the MQL are included in parentheses. Abbreviations: B, Berlin; H, Hessia; SW, surface water; RW, raw water; DW, drinking water; GW, groundwater. For details on samples, see Table S3. Note the logarithmic concentration scale

The method was applied to six samples relevant to drinking water production from Berlin and Hessia, Germany (surface water, groundwater, unventilated raw water, and finished drinking water; for sample details, see ESM Table S3). Quantification was performed based on correction factors given in ESM Table S6, which were determined together with the six samples, and the results are presented in Fig. 5 and ESM Table S8. Of the 15 target compounds, 9 were detected in at least one of the samples from Berlin and Hessia and 4 PM substances were found in at least four samples. Six compounds were even found in the final drinking water. This is underlining the importance of accurate quantification methods for PM substances as a crucial prerequisite for future risk assessment. For most of the compounds, the results indicated low concentrations in the water samples, typically below 100 ng L^−1^. However, ACE and TFMSA both exceeded 10 μg L^−1^ in a groundwater sample from Hessia. These findings are in agreement with the few reports from the literature on PM substances [7, 31, 32]. HHTMP, MEL, and 3,4-DMBSA (also reported by Betowski et al. [33]) and DPG (also reported by Tang et al. [34]) exceeded 100 ng L^−1^ in single samples but did not reach μg L^−1^ concentrations in our limited sample set.

## Conclusions

A rapid trace analytical method for the simultaneous quantification of 15 target PM substances (log *D* values − 3.06 to 1.23) was developed for water samples. The method is based on azeotrope evaporation of the samples, SFC-HRMS analysis, and external quantification using a correction factor for apparent recoveries. Trueness of quantification revealed results within ± 30% of the theoretical value in 77% of quantifications. To further increase accuracy, we recommend synthesis of isotope-labeled internal standards for the most important PM substances. This is the first method specifically designed for PM substances that does not include sample-specific calibration curves (standard addition quantification). The method is thus well suitable for large screening and monitoring programs. The method is generic and can easily be expanded to include further target PM substances or used in non-target or suspect screening of highly polar contaminants in water samples.

## Electronic supplementary material

ESM 1 (DOCX 394 kb)

## References

[1] Reemtsma T, Berger U, Arp HPH, Gallard H, Knepper TP, Neumann M, Quintana JB, de Voogt P (2016). Mind the gap: persistent and mobile organic compounds – water contaminants that slip through. Environ Sci Technol.

[2] Arp HPH, Brown TN, Berger U, Hale SE (2017). Ranking REACH registered neutral, ionizable and ionic organic chemicals based on their aquatic persistency and mobility. Environ Sci: Processes Impacts.

[3] Schulze S, Sättler D, Neumann M, Arp HPH, Reemtsma T, Berger U (2018). Using REACH registration data to rank the environmental emission potential of persistent and mobile organic chemicals. Sci Total Environ.

[4] Neumann M, Schliebner I. Protecting the sources of our drinking water: the criteria for identifying persistent, mobile and toxic (PMT) substances and very persistent and very mobile (vPvM) substances under EU Regulation REACH (EC) No 1907/2006. German Environment Agency (UBA Texte 127/2019). Dessau-Rosslau (87 pages, ISSN 1862-4804); 2019.

[5] Zahn D, Neuwald IJ, Knepper TP. Analysis of mobile chemicals in the aquatic environment – current capabilities, limitations and future perspectives. Anal Bioanal Chem. 2020. 10.1007/s00216-020-02520-z. 10.1007/s00216-020-02520-z 32086538

[6] Bruzzoniti MC, de Carlo RM, Sarzanini C (2008). Determination of sulfonic acids and alkylsulfates by ion chromatography in water. Talanta..

[7] Zahn D, Fromel T, Knepper TP (2016). Halogenated methanesulfonic acids: a new class of organic micropollutants in the water cycle. Water Res.

[8] Boulard L, Dierkes G, Ternes T (2018). Utilization of large volume zwitterionic hydrophilic interaction liquid chromatography for the analysis of polar pharmaceuticals in aqueous environmental samples: benefits and limitations. J Chromatogr A.

[9] Montes R, Aguirre J, Vidal X, Rodil R, Cela R, Quintana JB (2017). Screening for polar chemicals in water by trifunctional mixed-mode liquid chromatography-high resolution mass spectrometry. Environ Sci Technol.

[10] Montes R, Rodil R, Cela R, Quintana JB (2019). Determination of persistent and mobile organic contaminants (PMOCs) in water by mixed-mode liquid chromatography–tandem mass spectrometry. Anal Chem.

[11] Schulze S, Zahn D, Montes R, Rodil R, Quintana JB, Knepper TP, Reemtsma T, Berger U (2019). Occurrence of emerging persistent and mobile organic contaminants in European water samples. Water Res.

[12] Laboureur L, Guérineau V, Auxilien S, Yoshizawa S, Touboul D (2018). Profiling of modified nucleosides from ribonucleic acid digestion by supercritical fluid chromatography coupled to high resolution mass spectrometry. J Chromatogr A.

[13] Klesper E, Corwin AH, Turner DA (1962). High pressure gas chromatography above critical temperatures. J Organomet Chem.

[14] Saito M (2013). History of supercritical fluid chromatography: instrumental development. J Biosci Bioeng.

[15] Taylor LT (2009). Supercritical fluid chromatography for the 21st century. J Supercrit Fluids.

[16] Lesellier E (2009). Retention mechanisms in super/subcritical fluid chromatography on packed columns. J Chromatogr A.

[17] West C. How good is SFC for polar analytes? Chromatogr Today. 2013:22–9.

[18] Bieber S, Greco G, Grosse S, Letzel T (2017). RPLC-HILIC and SFC with mass spectrometry: polarity-extended organic molecule screening in environmental (water) samples. Anal Chem.

[19] Salvatierra-Stamp V, Ceballos-Magaña SG, Gonzalez J, Ibarra-Galván V, Muñiz-Valencia R (2015). Analytical method development for the determination of emerging contaminants in water using supercritical-fluid chromatography coupled with diode-array detection. Anal Bioanal Chem.

[20] Sen A, Knappy C, Lewis MR, Plumb RS, Wilson ID, Nicholson JK, Smith NW (2016). Analysis of polar urinary metabolites for metabolic phenotyping using supercritical fluid chromatography and mass spectrometry. J Chromatogr A.

[21] Parr MK, Wuest B, Naegele E, Joseph JF, Wenzel M, Schmidt AH, Stanic M, de la Torre X, Botrè F (2016). SFC-MS/MS as an orthogonal technique for improved screening of polar analytes in anti-doping control. Anal Bioanal Chem.

[22] Desfontaine V, Capetti F, Nicoli R, Kuuranne T, Veuthey JL, Guillarme D (2018). Systematic evaluation of matrix effects in supercritical fluid chromatography versus liquid chromatography coupled to mass spectrometry for biological samples. J Chromatogr B.

[23] Reemtsma T, Alder L, Banasiak U (2013). A multimethod for the determination of 150 pesticide metabolites in surface water and groundwater using direct injection liquid chromatography-mass spectrometry. J Chromatogr A.

[24] Köke N, Zahn D, Knepper TP, Frömel T (2018). Multi-layer solid-phase extraction and evaporation-enrichment methods for polar organic chemicals from aqueous matrices. Anal Bioanal Chem.

[25] Khalikova MA, Lesellier E, Chapuzet E, Šatínský D, West C (2018). Development and validation of ultra-high performance supercritical fluid chromatography method for quantitative determination of nine sunscreens in cosmetic samples. Anal Chim Acta.

[26] Abrahamsson V, Sandahl M (2013). Impact of injection solvents on supercritical fluid chromatography. J Chromatogr A.

[27] Tran NH, Hu J, Ong SL (2013). Simultaneous determination of PPCPs, EDCs, and artificial sweeteners in environmental water samples using a single-step SPE coupled with HPLC-MS/MS and isotope dilution. Talanta..

[28] Svan A, Hedeland M, Arvidsson T, Pettersson CE (2018). The differences in matrix effect between supercritical fluid chromatography and reversed phase liquid chromatography coupled to ESI/MS. Anal Chim Acta.

[29] Birch GF, Drage DS, Thompson K, Eaglesham G, Mueller JF (2015). Emerging contaminants (pharmaceuticals, personal care products, a food additive and pesticides) in waters of Sydney estuary Australia. Mar Pollut Bull.

[30] Perkola N, Sainio P (2014). Quantification of four artificial sweeteners in Finnish surface waters with isotope-dilution mass spectrometry. Environ Pollut.

[31] Buerge IJ, Buser HR, Kahle M, Müller MD, Poiger T (2009). Ubiquitous occurrence of the artificial sweetener acesulfame in the aquatic environment. Environ Sci Technol.

[32] Ruff M, Mueller MS, Loos M, Singer HP (2015). Quantitative target and systematic non-target analysis of polar organic micro-pollutants along the river Rhine using high-resolution mass-spectrometry – identification of unknown sources and compounds. Water Res.

[33] Betowski L, Kendall D, Donnelly C (1996). Characterization of groundwater samples from superfund sites by gas chromatography/mass spectrometry and liquid chromatography/mass spectrometry. Environ Sci Technol.

[34] Tang J, Tang L, Zhang C, Zeng G, Deng Y, Dong H, Wang J, Wu Y (2015). Different senescent HDPE pipe-risk: brief field investigation from source water to tap water in China (Changsha City). Environ Sci Pollut Res Int.

