# Supplementary material for: A rapid method for quantification of persistent and mobile organic substances in water using supercritical fluid chromatography coupled to high-resolution mass spectrometry
Source: Anal Bioanal Chem. 2020 Jun 10;412(20):4941–52. doi: 10.1007/s00216-020-02722-5 (PMC7334264; doi:10.1007/s00216-020-02722-5)
Supplement: Supplementary file 1 — (DOCX 394 kb) [file 216_2020_2722_MOESM1_ESM.docx]

**Electronic Supplementary Material**

**A rapid method for quantification of persistent and mobile organic substances in water using supercritical fluid chromatography coupled to high resolution mass spectrometry**

Stefanie Schulze^1^, Heidrun Paschke^1^, Till Meier^1^, Matthias Muschket^1^, Thorsten Reemtsma^1,2^, Urs Berger^1,*^

^1^ Helmholtz Centre for Environmental Research – UFZ, Department of Analytical Chemistry, Permoserstrasse 15, 04318 Leipzig, Germany

^2^ University of Leipzig, Institute of Analytical Chemistry, Linnéstrasse 3, 04103 Leipzig, Germany

* Corresponding author e-mail, urs.berger@ufz.de; phone, +49 341 235 4654; fax, +49 341 235 450822**Table of contents**

**Table S1.** List of target compounds sorted by log*D* (estimated at pH 7.0) S3

**Table S2.** Chemicals and instrumentation for the enrichment and for the instrumental methods S6

**Table S3.** Sample description S7

**Figure S1.** Gradient profiles of the mobile phases as a function of time S8

**Table S4.** SFC-HRMS mass spectrometric parameters and retention factors in chromatography S9

**Table S5.** RPLC-MS/MS mass spectrometric parameters and retention factors in chromatography S10

**Table S6.** Apparent recovery experiments: Spiking levels, mean apparent recoveries (mean of the different spiking levels and of the experiments in DW and SW) and the corresponding correction factor for analyte quantification S11

**Table S7.** Accuracy experiments: Spiking levels, precision (n=4) and trueness (mean value of n=4) for analyte quantification in the spiked river Mulde and drinking water sample S12

**Table S8.** Concentrations of PM substances quantified in water samples (Table S3) S13

**Table S1.** List of target compounds sorted by log*D* (estimated at pH 7.0)^1^

| **Analyte index** | **CAS registry number** | **Substance name** | **log*D*  (pH 7.0)^1^** | **Molecular weight**  **(g mol^-1^)** | **Formula** | **Structure  (main structure at pH 7.0)^1^** | **Supplier** | **Chemical standard grade** |
| --- | --- | --- | --- | --- | --- | --- | --- | --- |
| ACE | 55589-62-3 | Acesulfame (K) | -3.06 | 162.14 | C4H4NO4S |  | Sigma Aldrich | ≥ 99% |
| AAMPS | 5165-97-9 | 2-Acrylamino-2-methylpropane sulfonate (sodium salt) | -2.71 | 206.24 | C7H12NO4S |  | abcr GmbH |  |
| HHTMP | 52722-86-8 | 4-Hydroxy-1-(2-hydroxyethyl)-2,2,6,6-tetramethylpiperidine | -2.62 | 201.31 | C11H23NO2 |  | abcr GmbH | 98% |
| BTMA | 56-93-9 | Benzyltrimethylammonium (chloride) | -2.24 | 150.24 | C10H16N |  | Sigma Aldrich | 97% |
| MPSA | 1561-92-8 | 2-Methyl-2-propene-1-sulfonate (sodium salt) | -2.21 | 135.16 | C4H7O3S |  | Sigma Aldrich | 98% |
| MEL | 108-78-1 | Melamine | -2.02 | 127.13 | C3H7N6 |  | Sigma Aldrich | analytical standard |
| DMPMA | 5205-93-6 | *N*-(3-(Dimethylamino)-propyl)methacrylamide | -1.85 | 170.26 | C9H18N2O |  | Fluorochem |  |
| ATA | 768-94-5 | Adamantan-1-amine | -1.49 | 151.25 | C10H17N |  | Fluorochem |  |
| TFMSA | 1493-13-6 | Trifluoromethanesulfonic acid | -1.23 | 149.06 | CF3O3S |  | Sigma Aldrich | ≥ 99 % |
| DMSP | 3965-55-7 | Dimethyl 5-sulfoisophthalate (sodium salt) | -1.22 | 273.24 | C10H9O7S |  | Alfa Aesar | 98% |
| CG | 461-58-5 | Cyanoguanidine | -1.03 | 84.08 | C2H4N4 |  | Acros Organics | 99.5% |
| TSA | 104-15-4 | *p*-Toluenesulfonic acid | -0.71 | 171.19 | C7H7O3S |  | MP Biomedicals | ≥ 99% |
| SAC | 81-07-2 | Saccharin | -0.49 | 182.17 | C7H4NO3S |  | Sigma Aldrich | ≥ 99% |
| 3,4-DMBSA | 1300-72-7 | 3,4-Dimethylbenzenesulfonic acid | -0.20 | 185.22 | C8H9O3S |  | Sigma Aldrich |  |
| 2,3-DMBSA | 25321-41-9 | 2,3-Dimethylbenzenesulfonic acid | -0.20 | 185.22 | C8H9O3S |  | Sigma Aldrich |  |
| DCHSS | 23386-52-9 | Dicyclohexyl sulfosuccinate (sodium salt) | 0.42 | 361.43 | C16H25O7S |  | Sigma Aldrich | ≥ 98% |
| DPG | 102-06-7 | 1,3-Diphenylguanidine | 1.23 | 212.28 | C13H14N3 |  | Sigma Aldrich | 97% |

^1^ Calculated using ChemAxon (<https://www.chemaxon.com/download/jchem-for-office/#jc4x>)

**Table S2.** Chemicals and instrumentation for the enrichment and for the instrumental methods

|  | **Chemicals and instrumentation** |
| --- | --- |
| **Enrichment method** |  |
| Evaporation (AZEVAP) | *Instrumentation:* Evaporator system XcelVap (Biotage, Uppsala, Sweden)  *Chemicals:* Glas Fibre Filters Whatman (GE Healthcare, Freiburg, Germany)  Acetonitrile (Biosolve, Valkenswaard, Netherlands) |
| **Instrumental methods** |  |
| SFC | *Instrumentation:*  *SFC:* Acquity UPC^2^ (Waters)  *Column:* Waters Acquity UPC^2^ BEH 3.0 x 100 mm, 1.7 µm Waters Acquity UPC^2^ Torus Diol 3.0 x 100 mm, 1.7 µm Waters Acquity UPC^2^ BEH-2EP 3.0 x 100 mm, 1.7 µm Waters Acquity UPC^2^ Torus-2Pic 3.0 x 100 mm, 1.7 µm  *Mass Spectrometer:* Synapt G2S (Waters)  *Software:* MassLynx (Waters)  *Chemicals:*  Carbon dioxide (Air Products, Pennsylvania, USA)  Methanol (Biosolve, Valkenswaard, Netherlands)  Ammonium hydroxide (~25% in water, Biosolve, Valkenswaard, Netherlands)  Formic acid, ammonium acetate, ammonium formate (Sigma Aldrich, Taufkirchen, Germany) |
| RPLC | *Instrumentation:* *UHPLC:* Acquity i-Class (Waters)  *Column:* Waters Acquity UPLC HSS T3, 2.1 x 50 mm, 1.8 µm  *Mass Spectrometer*: Xevo TQ-S (Waters)  *Software*: MassLynx (Waters)  *Chemicals:*  Methanol, ammonium formate (Biosolve, Valkenswaard, Netherlands) |

**Table S3.** Sample description

|  | **Sample name** | **Collection date** | **Sample type** | **Depth** | **Location** | **Connections between the samples** |
| --- | --- | --- | --- | --- | --- | --- |
| **Method development** | River Götsche | 06.12.2017 | Surface water, small stream | - | Saxony Anhalt (Germany) | - |
|  | River Mulde | 01.04.2019 | Surface water, river | - | Saxony Anhalt (Germany) |  |
|  | Drinking water | 06.12.2017 and 30.07.2019 | Tap water | - | Our laboratory |  |
| **Method application** | B-SW-1 | 27.10.2017 | Surface water from Tegeler See | - | Berlin (Germany) | B-SW is the source water for B-RW-1 |
|  | B-RW-1 | 26.10.2017 | Unventilated raw water from drinking water treatment plant Tegel | - | Berlin (Germany) | B-SW is the source water for B-RW-1 |
|  | B-DW-1 | 26.10.2017 | Finished drinking water from drinking water treatment plant Tegel | - | Berlin (Germany) | B-DW-1 is the finished drinking water made of B-SW-1 via B-RW-1 |
|  | H-GW-1 | 23.10.2017 | Groundwater of a well | 15 m | South Hessia (Germany) | - |
|  | H-GW-2 | 24.10.2017 | Groundwater of a well | 30 m | South Hessia (Germany) | H-GW-2 is the raw water for H-DW-2 |
|  | H-DW-2 | 24.10.2017 | Drinking water | - | South Hessia (Germany) | H-DW-2 is the finished drinking water made of H-GW-2 |

**
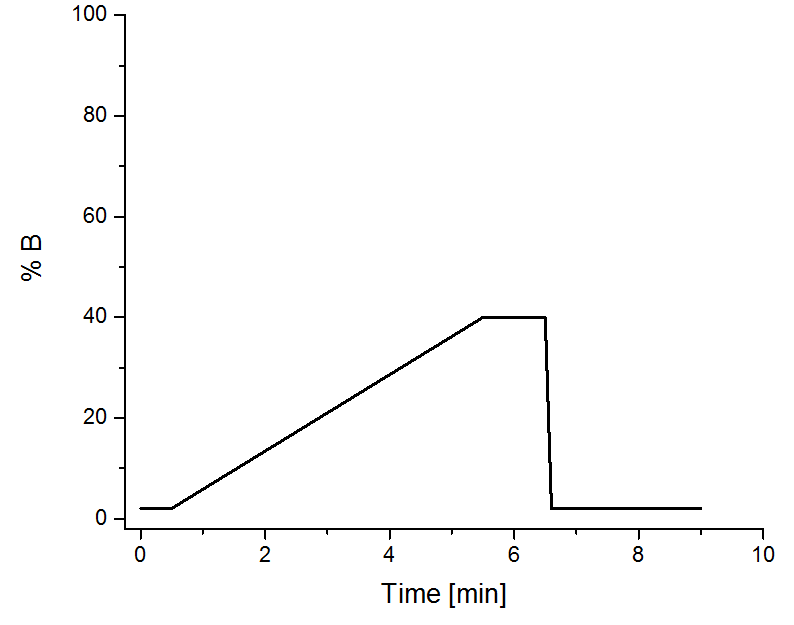
A**

**
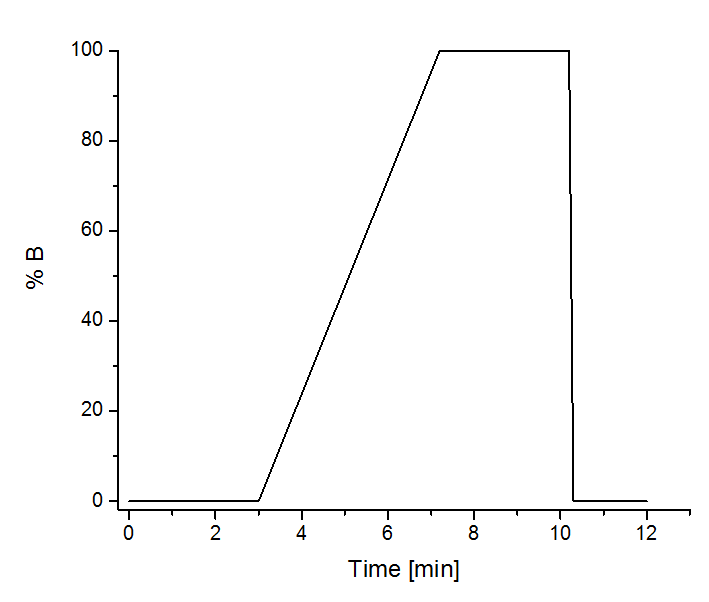
**

**B**

Figure S1. Gradient profiles of the mobile phases as a function of time for A) SFC on an Acquity UPC^2^ BEH or Torus Diol column (same gradient used); solvent A: CO_2_; (co-)solvent B: MeOH/H_2_O 95/5, 0.2 % NH_4_OH; make-up flow: 0.3 mL min^-1^ MeOH/H_2_O 90/10, 0.1 % HCOOH, pH 6; B) RPLC on an Acquity UPLC HSS T3 column; solvent A: H_2_O, 5 mM NH_4_COOH; solvent B: MeOH, 5 mM NH_4_COOH.

**Table S4.** SFC-HRMS mass spectrometric parameters (see also footnote) and retention factors in chromatography

| **Analyte** | **ESI mode** | **Quantifyer *m/z*** | **Qualifyer *m/z*** | **Retention factor k’**  **(BEH)** | **Retention factor k’**  **(Torus Diol)** |
| --- | --- | --- | --- | --- | --- |
| ACE | neg | 161.99 | 82.03 | 12.6 | 14.0 |
| AAMPS | neg | 206.05 | 135.01 | 13.7 | 14.1 |
| HHTMP | pos | 202.18 | 102.09 | 12.6 | 10.0 |
| BTMA | pos | 150.13 | 91.06 | 12.8 | 13.7 |
| MPSA | neg | 135.01 | - | 12.4 | 13.1 |
| MEL | pos | 127.07 | 85.05 | 12.5 | 14.0 |
| DMPMA | pos | 171.15 | 126.09p | 14.2 | 9.6 |
| ATA | pos | 152.14 | 134.11 | 12.3 | 11.2 |
| TFMSA | neg | 148.95 | 79.96 | 12.1 | 13.1 |
| DMSP | neg | 273.01 | 209.05 | 12.7 | 13.7 |
| CG | pos | 85.05 | - | 10.4 | 9.6 |
| TSA | neg | 171.01 | 107.05 | 12.8 | 14.1 |
| SAC | neg | 181.99 | 105.96 | 13.0 | 14.8 |
| 3,4-/2,3-DMBSA | neg | 185.03 | 121.07 | 12.5 / 12.7 | 13.3 / 13.6 |
| DCHSS | neg | 361.13 | 80.97 | 11.7 | 12.2 |
| DPG | pos | 212.12 | 195.09 | 12.1 | 11.6 |

*ESI-HRMS parameters:* Full Scan recording *m/z* 50 to 600; Analyser Mode Resolution; Scan time 0.08 s; Cone Voltage 20 kV; Capillary 700 V (+) / 2000 V (-); Source Temperature 140 °C; Desolvation Temperature 550 °C; Function 1: Trap Collision Energy 4 eV, Transfer Collision Energy 2 eV; Function 2: Trap Collision Energy 15 eV, Transfer Collision Energy 40 eV; Function 3 (Lock Spray Configuration): Reference Cone Voltage 30 V, Reference Trap Collision Energy 4 eV, Lock Mass (leucine enkephaline) 556.2771 (+) / 554.2615 (-); k’ = (t_R_-t_0_)/t_0_, t_R_ – retention time of the analyte, t_0_ – void time.

**Table S5.** RPLC-MS/MS mass spectrometric parameters (see also footnote) and retention factors in chromatography

| **Analyte** | **ESI mode** | **Q1 *m/z*** | **Q2 *m/z*** | **Cone [V]** | **Collision energy [eV]** | **Retention factor**  **k’** |
| --- | --- | --- | --- | --- | --- | --- |
| ACE | neg | 162 | 78 | 4 | 22 | 1.8 |
| ACE | neg | 162 | 82 | 4 | 14 | 1.8 |
| AAMPS | neg | 206 | 80 | 48 | 24 | 4.6 |
| AAMPS | neg | 206 | 135 | 48 | 18 | 4.6 |
| HHTMP | pos | 202 | 62 | 16 | 16 | 3.4 |
| HHTMP | pos | 202 | 102 | 16 | 16 | 3.4 |
| BTMA | pos | 150 | 58 | 2 | 14 | 9.4 |
| BTMA | pos | 150 | 91 | 2 | 20 | 9.4 |
| MPSA | neg | 135 | 80 | 58 | 14 | 1.4 |
| MPSA | neg | 135 | 91 | 58 | 8 | 1.4 |
| MEL | pos | 127 | 43 | 2 | 20 | 0.6 |
| MEL | pos | 127 | 85 | 2 | 16 | 0.6 |
| DMPMA | pos | 171 | 69 | 2 | 22 | 5.4 |
| DMPMA | pos | 171 | 126 | 2 | 12 | 5.4 |
| ATA | pos | 152 | 93 | 38 | 24 | 19 |
| ATA | pos | 152 | 135 | 38 | 16 | 19 |
| TFMSA | neg | 149 | 80 | 4 | 18 | 0.8 |
| TFMSA | neg | 149 | 99 | 4 | 18 | 0.8 |
| DMSP | neg | 273 | 150 | 26 | 26 | 18 |
| DMSP | neg | 273 | 209 | 26 | 22 | 18 |
| CG | pos | 85 | 41 | 60 | 14 | 0.4 |
| CG | pos | 85 | 68 | 60 | 12 | 0.4 |
| TSA | neg | 171 | 80 | 2 | 26 | 0.6 |
| TSA | neg | 171 | 107 | 2 | 20 | 0.6 |
| SAC | neg | 182 | 42 | 4 | 18 | 1 |
| SAC | neg | 182 | 106 | 4 | 18 | 1 |
| 3,4-/2,3-DMBSA | neg | 185 | 80 | 6 | 24 | 0.5 / 0.7 |
| 3,4-/2,3-DMBSA | neg | 185 | 121 | 6 | 22 | 0.5 / 0.7 |
| DCHSS | neg | 361 | 81 | 6 | 22 | 23.5 |
| DCHSS | neg | 361 | 197 | 6 | 24 | 23.5 |
| DPG | pos | 212 | 77 | 28 | 32 | 19.2 |
| DPG | pos | 212 | 119 | 28 | 20 | 19.2 |

*ESI-MS/MS parameters:* Capillary Voltage 1000/1420 V (+/-); Source Temperature 150 °C; Desolvation Temperature 600 °C; Cone Gas Flow 150 L h^-1^; Collision Gas Flow 0.15 mL min^-1^; Nebuliser Gas Pressure 100 psi; k’ = (t_R_-t_0_)/t_0_, t_R_ – retention time of the analyte, t_0_ – void time.

**Table S6.** Apparent recovery experiments: Spiking levels, mean apparent recoveries (mean of the different spiking levels and of the experiments in DW and SW) and the corresponding correction factor for analyte quantification

| **Analyte** | Spiking levels [ng L^-1^] | Mean apparent recovery [%] | Correction factor for quantification |
| --- | --- | --- | --- |
| ACE | 50 / 250 / 5000 | 42 | 2.40 |
| AAMPS | 50 / 300 | ^1^ | ^1^ |
| HHTMP | 50 / 300 | 51 | 1.96 |
| BTMA | 10 / 50 / 300 | 85 | 1.18 |
| MPSA | 100 /500 | 21 | 4.76 |
| MEL | 50 / 250 | 81 | 1.23 |
| DMPMA | 10 / 50 / 300 | 31 | 3.21 |
| ATA | 10 / 50 / 300 | 58 | 1.72 |
| TFMSA | 10 / 50 / 300 | 78 | 1.28 |
| DMPS | 50 / 300 | ^1^ | ^1^ |
| CG | 60 / 300 | 71 | 1.40 |
| TSA | 30 / 150 / 600 | 104 | 0.96 |
| SAC | 30 / 150 / 1200 | 97 | 1.04 |
| 3,4-DMBSA | 20 / 100 / 500 | 54 | 1.86 |
| 2,3-DMBSA | 100 / 500 | 88 | 1.14 |
| DCHSS | 50 / 300 | 36 | 2.81 |
| DPG | 100 / 500 | 61 | 1.64 |

^1^ Spiking levels too low to reliably determine the apparent recovery

Table S7. Accuracy experiments: Spiking levels, precision (*n*=4) and trueness (mean value of *n*=4) for analyte quantification in the spiked river Mulde and drinking water sample

|  | **Götsche river** | | | **Mulde river sample** | | | **Drinking water sample** | | |
| --- | --- | --- | --- | --- | --- | --- | --- | --- | --- |
| **Analyte** | Spiking level for determination of apparent recovery [ng L^-1^] | Apparent recovery [%] | Correction factor for quantification | Spiking level [ng L^-1^] | Precision (relative standard deviation *n*=4) [%] | Trueness (deviation from spiked concentration) [%] | Spiking level [ng L^-1^] | Precision (relative standard deviation *n*=4) [%] | Trueness (deviation from spiked concentration) [%] |
| ACE | 85,900 | 91 | 1.10 | 25,800 | 5 | 3 | 2,580 | 3 | -57 |
| HHTMP | 450 | 88 | 1.13 | 135 | 4 | -3 | 13.5 | 15 | 23 |
| BTMA | 6,880 | 72 | 1.40 | 2,060 | 10 | -16 | 206 | 15 | -27 |
| MPSA | 275,000 | 38 | 2.62 | 82,500 | 10 | 64 | 8,250 | 32 | -58 |
| MEL | ^1^ | ^1^ | ^1^ | ^1^ | 14^2^ | ^1^ | ^1^ | 22^2^ | ^1^ |
| DMPMA | 1,380 | 41 | 2.41 | 413 | 10 | -17 | 41.3 | 7 | 11 |
| ATA | 1,250 | 64 | 1.56 | 375 | 4 | 13 | 37.5 | 7 | -2 |
| TFMSA | 1,500 | 107 | 0.93 | 450 | 4 | 21 | 45.0 | 4 | 8 |
| CG | ^3^ | ^3^ | ^3^ | ^3^ | 14^2^ | ^3^ | ^3^ | 5^2^ | ^3^ |
| TSA | 22,800 | 61 | 1.64 | 6,830 | 7 | 13 | 683 | 16 | -29 |
| SAC | 196,000 | 72 | 1.39 | 58,900 | 5 | 28 | 5,890 | 3 | -38 |
| 3,4-DMBSA | 22,900 | 42 | 2.36 | 6,860 | 9 | 28 | 686 | 5 | 8 |
| 2,3-DMBSA | 22,900 | 72 | 1.40 | 6,860 | 14 | 5 | 686 | 5 | -19 |
| DCHSS | 2,250 | 29 | 3.49 | 675 | 10 | -25 | 67.5 | 2 | 65 |
| DPG | 325 | 84 | 1.19 | 97.5 | 7 | 1 | 9.75 | 6 | 73 |

^1^ Concentration already present in river Götsche water too hight to perform spiking experiments

^2^ Precision data from earlier apparent recovery experiments from Table S6 (*n*=3)

^3^ Linear range of detection too small to perform trueness experiments (see Table 1 in the main text)

**Table S8.** Concentrations of PM substances quantified in water samples (Table S3)

| **Analyte** | B-SW-1  [ng L^-1^] | B-RW-1  [ng L^-1^] | B-DW-1  [ng L^-1^] | H-GW-1  [ng L^-1^] | H-GW-2  [ng L^-1^] | H-DW-2  [ng L^-1^] |
| --- | --- | --- | --- | --- | --- | --- |
| ACE | 349 | 282 | <MQL | 70,200 | 8,210 | 989 |
| HHTMP | nd | nd | nd | nd | 107 | 25 |
| BTMA | nd | nd | nd | nd | nd | nd |
| MPSA | nd | nd | nd | nd | nd | nd |
| MEL | 233 | 13 | 33 | <MQL | nd | nd |
| DMPMA | nd | nd | nd | nd | nd | nd |
| ATA | 9 | 5 | 5 | 36 | nd | nd |
| TFMSA | nd | nd | nd | 18,800 | nd | nd |
| CG | nd | nd | nd | nd | nd | nd |
| TSA | <MQL | nd | nd | nd | nd | <MQL |
| SAC | 44 | nd | nd | 86 | nd | nd |
| 3,4-DMBSA | 141 | 43 | <MQL | 150 | nd | <MQL |
| 2,3-DMBSA | nd | nd | nd | nd | nd | nd |
| DCHSS | nd | nd | nd | nd | nd | nd |
| DPG | 191 | nd | nd | nd | nd | nd |

- Method not applicable
nd Not detected (<MDL)
<MQL Detected but not quantifyable
